# Trends in Gallbladder Disease in Young Adults: A Growing Concern

**DOI:** 10.7759/cureus.28555

**Published:** 2022-08-29

**Authors:** Farah Naaz Kazi, Shaurav Ghosh, J V Pranav Sharma, Shwetha Saravanan, Sanjana Patil

**Affiliations:** 1 Surgery, Medicine, Vydehi Institute of Medical Sciences and Research Centre, Bengaluru, IND; 2 General Surgery, Vydehi Institute of Medical Sciences and Research Centre, Bengaluru, IND; 3 Surgery, Vydehi Institute of Medical Sciences and Research Centre, Bengaluru, IND

**Keywords:** common bile duct, ercp, cholelithiasis, young, gall blader disease

## Abstract

Millennials (age: 25-32 years) and Generation-Z individuals (age: 10-25 years) exhibit a shift in the occurrence of gallbladder diseases, which may be related to changes in lifestyle and genetics. In light of these findings, we performed a retrospective observational study on patients who underwent gallbladder surgeries to determine the trend in gallbladder diseases in young adults. Both categorical and continuous data on 90 patients were collected between January 2020 and June 2021 and analysed retrospectively, with differences considered significant at a *p*-value of 0.05. The diagnosis of gallstones in young adults is presently complicated, as the signs and symptoms of biliary tract sickness differ significantly between those under and over 30 years of age. We observed that gallbladder diseases and their complications were highly common in individuals between the ages of 21 and 25 years. We discovered that gallstones were more common in teenagers than previously thought. Delays in intervention resulted in future complications which could have been avoided.

## Introduction

Although gallstones are considered to affect only ‘fair, fat, fertile, 40-year-old females,’ they can be reported at any age [1-11]. Although gallstone disease is common in young adults aged 20-40 years, its symptoms do not appear until later stages of life [2,3,8]. According to the Western literature, 4-20% of the reported series of cholecystectomies are performed on patients under the age of 30 years [11].

The unalterable risk factors associated with gallstones include gender (females are more prone than males), age (gallstones are mostly reported in adults), and ethnicity or family history (genetic traits). However, obesity, metabolic syndromes, rapid weight loss, diseases such as cirrhosis and Crohn’s disease, and gallbladder stasis are treatable. Nonetheless, a high caloric intake is the only known dietary risk. Diets rich in fibre, vegetable proteins, nuts, calcium, vitamin C, coffee, and alcohol, as well as physical activity, may prevent the formation of gallstones [1-4,11].

Asymptomatic cholelithiasis rarely requires surgery. However, patients with large (>2.50 cm) gallstones, congenital haemolytic anaemia, non-functioning gallbladders, or those undergoing bariatric surgery or colectomy warrant a prophylactic cholecystectomy. The symptomology of biliary tract disease varies significantly in patients under and over the age of 30 years. The male-to-female ratio is 1:5, and young patients are three to four times more prone to develop acute cholecystitis [11]. Pain in the epigastrium or the upper right quadrant 30-60 min after meals is frequently associated with gallstone disease [2]. The increased radiation in young patients confounds the diagnosis. However, in young patients, fatty-food intolerance and complaints of dyspepsia, eructation, and flatulence, as well as associated diseases are uncommon [1-3,11]. Patients under the age of 30 years undergo cholecystectomy with minimal morbidity and no mortality [11].

Recently, we observed an increase in the number of teenagers and young adults undergoing endoscopic retrograde management of common bile duct (CBD) stones at our facility. This may be attributed to an increase in the prevalence of symptomatic gallstone disease in this age group. Therefore, we conducted a retrospective observational study to determine the prevalence of gallbladder disease in the age group of 18-25 years.

## Materials and methods

Data were collected retrospectively from the records of the out-patient department (OPD) and operation theatre (OT) of the Department of General Surgery at the Vydehi Institute of Medical Sciences and Research Centre, Bangalore, between January 2020 and June 2021. We examined all complete abdominal ultrasounds of young adults that complained of abdominal pain and indicated follow-up of various abdominal pathologies, malformation screening, or urinary tract infection.

A total of 212 patients with gallbladder disease and pathology were identified, out of which 90 patients underwent surgery and were monitored regularly. We recorded the age of the patient at the time of admission, gender, gallbladder pathologies, and liver function tests (LFT).

Data were collated in a spreadsheet in Microsoft Excel (Microsoft, Redmond, WA, USA) and analysed using Statistical Package for Social Sciences (SPSS) version 22 (IBM Corp., Armonk, NY, USA). Categorical data were represented as frequencies and proportions, whereas continuous data were represented as means and standard deviations. Analysis of variance (ANOVA) was used to determine the significance of differences between more than two quantitative variables, and a p-value <0.05 was considered statistically significant.

## Results

Table 1 shows the distribution of patients according to age group. The majority (n = 51; 56.7%) of the patients were aged between 26 years and 30 years, with the minimum and maximum ages being 18 years and 30 years, respectively, and the mean age being 25.84 + 3.3 years. Approximately 34.4% of the patients (n = 31) were in the age group of 21-25 years.

**Table 1:** Distribution of subjects according to age group

Age distribution	Frequency(n)	Percent(%)
<20 years	08	8.90
21-25 years	31	34.40
26-30 years	51	56.70
Total	90	100.00

Table 2 shows the distribution of patients according to gender. Gallstones were mostly reported in females (n = 65; 72.2%) as opposed to males (n = 25; p = 7.8%) indicating a significant disparity. The main reason why women tend to be more affected by gallstones is due to the differences in hormone production between the sexes. Oestrogen and progesterone, which are produced by women, are the key hormones involved in this discrepancy. 

**Table 2:** Distribution of subjects according to sex

Sex Distribution	Frequency(n)	Percent(%)
Female	65	72.20
Male	25	7.80
Total	90	100.00

Table 3 shows the distribution of patients according to age and gallbladder pathologies. In this study, most of the patients in the age group of <20 years reported cholelithiasis and β-thalassemia (12.5% each). Furthermore, the prevalence of cholelithiasis and cholecystitis increased with age.

**Table 3:** Distribution of subjects according to age and gallbladder pathologies CBT: common bile duct, ERCP: endoscopic retrograde cholangiopancreatography, S/P: status post

Gallbladder pathology	<20 years		21–25 years		26–30 years	
	n	%	n	%	n	%
Acute calculous cholecystitis	0	0.00	0	0.00	1	2.00
Beta thalassemia with cholelithiasis	1	12.50	0	0.00	0	0.00
Calculous cholecystitis	0	0.00	2	6.40	0	0.00
Calculous cholelithiasis	0	0.00	0	0.00	1	2.00
Cholecystitis S/P partial cholecystectomy	0	0.00	1	3.20	0	0.00
Choledochal cyst other types	0	0.00	0	0.00	2	3.90
Choledochal cyst type 1	1	12.50	0	0.00	0	0.00
Choledochal cyst type 4A	0	0.00	0	0.00	1	2.00
Cholelithiasis	1	12.50	5	16.10	10	19.60
Cholelithiasis with choledocholithiasis	0	0.00	1	3.20	1	2.00
Chronic calculous cholecystitis with pseudocyst of pancreas	0	0.00	1	3.20	0	0.00
Chronic calculous cholelithiasis	0	0.00	4	12.90	5	9.80
Chronic calculus cholecystitis	0	0.00	12	38.70	21	41.20
Chronic cholecystitis	0	0.00	0	0.00	2	4.00
Chronic cholelithiasis	0	0.00	1	3.20	0	0.00
Dilated CBD with cholelithiasis	0	0.00	0	0.00	1	2.00
Gall bladder polyp	0	0.00	1	3.20	2	3.90
Gall bladder sludge with chronic cholecystitis	0	0.00	0	0.00	1	2.00
Obstructive jaundice and calculi in the CBD	0	0.00	0	0.00	1	2.00
S/P cholecystectomy with discharging sinus over the scar	0	0.00	1	3.20	0	0.00
S/P ERCP for choledocholithiasis	0	0.00	0	0.00	1	2.00
S/P ERCP for choledocholithiasis with cholelithiasis	0	0.00	1	3.20	0	0.00
S/P ERCP with cholelithiasis	0	0.00	0	0.00	1	2.00
Splenomegaly and chronic cholecystitis	0	0.00	1	3.20	0	0.00
Type 4 choledochal cyst	1	12.50	0	0.00	0	0.00

Table 4 shows the distribution of patients according to age and the results of LFTs. Patients in the age group of 21-25 years exhibited significantly high aspartate aminotransferase (AST), alanine transaminase (ALT) and alkaline phosphatase (ALP) with p- values of 0.544, 0.337 and 0.518 respectively. Moreover, we observed the maximum increase in total bilirubin (TB) in the patients in the age group of 26-30 years.

**Table 4:** Distribution of subjects according to age and liver function tests (LFTs) TB: total bilirubin, AST: aspartate aminotransferase, ALT: alanine transaminase, ALP: alkaline phosphatase

AGE GROUP		TB	AST	ALT	ALP
<20 years	Mean	1.82	27.38	30.00	93.63
	SD	2.37	8.14	16.49	25.59
21-25 years	Mean	1.22	55.32	68.06	121.39
	SD	1.65	67.99	88.47	135.66
26-30 years	Mean	2.28	43.57	50.63	94.55
	SD	7.24	73.41	64.43	90.85
Overall	Mean	1.88	46.18	54.80	103.71
	SD	5.57	68.24	71.54	105.16
P Value		0.70	0.54	0.34	0.52

## Discussion

According to the study by Sirmione [1], women in the age group of 18-65 years are more prone to gallstone disease compared to men in the same age group, with an overall cholelithiasis prevalence of 6.9% (8.9% for women and 4.5% for men). Furthermore, the frequency of gallbladder disease increases with age irrespective of the gender of the patient. Biliary pain was reported by 22% of the patients with gallstones. However, 108 (82%) of the 132 patients with gallstones were unaware of their condition before the study.

Previous reports suggest that the likelihood of developing gallstone disease increases with the number of pregnancies and disorders such as obesity and hypertriglyceridemia [1-4]. Schirmer et al. (2005) concluded that approximately 20% of individuals over 40 years and 30% of those over 70 years of age are likely to develop biliary calculi. During the reproductive phase, the proportion increases approximately four times in women compared to men, with the gender gap reaching approximately equality with age [2]. In this study, 34.4% of the patients belonged to the age group of 21-25 years (Table 1), and this proportion increased with age. Women constituted 72.2% of the sample (90 patients) (Table 2) and hence the male-to-female ratio was 1:4. Although gallbladder stones were present in young adults, they exhibited symptoms, which became complicated with age [2,3].

According to Shaffer, the risk factors associated with gallstones include female gender, advancing age, and ethnic background [3]. The findings of our study corroborated these findings. In a 2008 retrospective analysis, the frequency of idiopathic gallstone disease and associated complications was higher than those reported in previous investigations [4]. In this study, 8.9% of the patients were < 20 years old (Table 1) and exhibited increased TB and activity of liver enzymes (Table 4). This indicates that an interdisciplinary approach using multiple strategies is necessary to manage gallbladder stones [4].

According to a 2012 population-based, cross-sectional study on 510,816 patients aged between 10 years and 19 years, the increase in childhood obesity increased the prevalence of gallstone disease in children and adolescents [5]. A retrospective, cross-sectional study conducted in 2012 by Mehta et al. on 404 children < 18 years of age revealed a statistically significant link between obesity and symptomatic juvenile gallbladder disease [6]. Another study revealed that idiopathic gallstone disease and its complications were more common than those predicted by previous studies [3,4]. Owing to the complexity of the disease, adolescent girls with abdominal pain and idiopathic gallstones require special care [4]. Moreover, to emphasise the significant prevalence of gallstones and their pathogenesis in young people, Constantinescu et al. examined 1,905 cholecystectomies and indicated that age, female gender, pregnancy, and obesity were the most common risk factors associated with gallstones [7]. This was verified by a Korean study [10]. 

In this study, the prevalence of cholelithiasis and cholecystitis increased with age (Table 3). Furthermore, as a result of delayed interventions, the rate of complications increased proportionately. Duct stones and severe pancreatitis were reported as frequent side-effects of gallstones in younger patients [7].

In a retrospective study on 574 patients with cholelithiasis, Chilimuri et al. revealed that the prevalence of cholelithiasis in young patients increased to 2.96% in 2010 within 15 years, with a 30% increase in the number of individuals with gallstone disease who needed hospitalisation. This pattern was highly evident in females [8]. However, gallbladder stones in young age groups could not be undetected in this study because they were asymptomatic [7,8]. Nonetheless, the LFT values, particularly the activities of specific liver enzymes, increased with age, similar to complications. This finding was similar to that of our study (Table 4). Patients aged 21-25 years exhibited high levels of liver enzymes, whereas those aged 26-30 years exhibited high levels of total bilirubin.

Therefore, we concluded that the likelihood of gallstone formation and its complications increased with age. Hence, all teenagers with gallstones must undergo early cholecystectomy because symptomatic idiopathic gallstones in adolescents are associated with an increased incidence of pancreatitis and CBD blockage [9]. 

This study does have some limitations. First, we focused on teenagers and young adults in our study. To raise the study's degree of focus, the participants' average age must be lowered. To obtain a focused larger sample size, necropsy findings and imaging investigations of this age range must be taken into consideration. Second, it seemed like our study had a limited sample size. Our study might have produced more accurate results if it had been based on a bigger sample size. Compared to qualitative investigations, the significance of sample size is higher for quantitative studies.

The main drawback of ultrasound in the imaging of diseases associated with gallstones is its frequent inability to evaluate the distal CBD because of surrounding bowel gas. The presence of a CBD stone can be determined by visualising the proximal biliary dilatation and by observing the patient's clinical symptoms, such as unpleasant jaundice or an obstructive pattern on liver function tests. This necessitates additional testing using various imaging techniques, increasing the expense and restricting the number of participants who can undergo additional testing.

## Conclusions

Our study has indicated that biliary tract sickness was significantly altered in the 90 patients under and over 30 years of age post gallbladder surgeries. Young female adults were more affected and the frequency of gallbladder disease and its complications increased in people between the ages of 21 years and 25 years. However, the diagnosis was complicated because of the various clinical symptoms. Young patients rarely exhibited dyspepsia, belching, flatulence, or an aversion to fatty foods which are the textbook symptoms of gallstones. Based on these findings, we concluded that routine screening of young adults is necessary to detect gallbladder diseases and their management requires an interdisciplinary approach with several interventions. Given the increased incidence of gallbladder stones in teenagers and younger adults, additional study is needed to examine epidemiological patterns and gain insight into the precise aetiology, genetics, and prognosis of gallbladder disease.
